# Infertility and Adenomyosis

**DOI:** 10.1155/2012/786132

**Published:** 2011-12-26

**Authors:** Sebastiano Campo, Vincenzo Campo, Giuseppe Benagiano

**Affiliations:** ^1^Institute of Obstetrics and Gynaecology, Catholic University of Sacred Heart, Largo Agostino Gemelli, 00168 Roma, Italy; ^2^Department of Gynaecology, Obstetrics and Urology, Sapienza University of Rome, Policlinico Umberto I, 00161 Roma, Italy

## Abstract

Classically, the diagnosis of adenomyosis has only been possible on a hysterectomy specimen, usually in women in their late fourth and fifth decades, and, therefore, evaluating any relationship with infertility was simply not possible. As a consequence, to this day, no epidemiologic data exists linking adenomyosis to a state of subfertility. Today, new imaging techniques have enabled a noninvasive diagnosis at a much earlier time and a number of single-case or small series reports have appeared showing that medical, surgical, or combined treatment can restore fertility in women with adenomyosis, an indirect proof of an association. At the functional level, several anomalies found in the so-called junctional zone, or inner myometrium, in adenomyosis patients have been shown to be associated with poor reproductive performance, mainly through perturbed uterine peristalsis. Additional evidence for an association comes from experimental data: in baboons, adenomyosis is associated with lifelong primary infertility, as well as to endometriosis. Finally, indirect proof comes from studies of the eutopic and ectopic endometrium in women with adenomyosis proving the existence of an altered endometrial function and receptivity. In conclusion, sufficient indirect proof exists linking adenomyosis to infertility to warrant systematic clinical studies.

## 1. Introduction

Adenomyosis has been defined as the “benign invasion of endometrium into the myometrium, producing a diffusely enlarged uterus which microscopically exhibits ectopic nonneoplastic endometrial glands and stroma surrounded by a hypertrophic and hyperplastic myometrium” [1]. Two separate pathogenetic theories have been advanced to explain its formation: an origin from the invagination of the deepest portion of the endometrial mucosa between bundles of smooth muscle fibres of the myometrium, or along the intramyometrial lymphatic system; a metaplastic process initiating from ectopic intramyometrial endometrial tissue produced *de novo* [2].

It has long been suspected that the presence of adenomyosis provokes a condition of subfertility. Unfortunately, unlike endometriosis where an association with infertility has been all but proven [3], classically the diagnosis of adenomyosis has been, until recently, carried out on hysterectomy specimens and in women in their late thirties and forties. This reality made it impossible to evaluate its effects on fertility [4].

Nonetheless, as early as 1988, Honoré et al. [5] published three cases of “adenomyoma,” a rare, localised form of adenomyosis [6], in young infertile women in whom surgery was carried out because of a diagnosis of leiomyoma. Based on this finding, they advocated early diagnosis and surgical intervention.

The situation changed some 25 years ago, with the identification through magnetic resonance (MR) imaging of a new functional uterine zone: the junction between the endometrium and the inner myometrium, named uterine junctional zone (JZ), measuring, in healthy young women, 5 mm in thickness or less [7]; this zone is clearly thickened in the presence of adenomyosis [8]. This was followed by attempts at identifying both the JZ and the presence of adenomyosis through ultrasonography [9], a technique now validated using a coronal section of the uterus obtained by three-dimensional trans vaginal sonography (TVS) [10].

Today both techniques can be utilised for an accurate evaluation and measurement of the JZ and of its alterations in the presence of adenomyosis, since they have good sensitivity and specificity. With regard to TVS, in a histologically controlled study Exacoustos et al. [10] have shown that the presence of myometrial cysts represents the most specific 2D-TVS feature for a correct diagnosis of adenomyosis, with a specificity of 98% and an accuracy of 78%. In their study, the most sensitive feature was the presence of a heterogeneous myometrium (sensitivity: 88%; accuracy: 75%). For 3D-TVS the best sensitivity is given by a JZ difference in thickness ≥4 mm and JZ infiltration and distortion (88%), with an accuracy of 85% and 82%, respectively. Exacoustos et al. concluded: “for 2D-TVS and 3D-TVS, respectively, the overall accuracy for diagnosis of adenomyosis was 83% and 89%, the sensitivity was 75% and 91%, the specificity was 90% and 88%, the positive predictive value was 86% and 85% and the negative predictive value was 82% and 92%.”

According to Dueholm et al. [11], MR imaging is superior to TVS for the diagnosis of adenomyosis, having equal sensitivity but a higher specificity (sensitivity: MR 0.70 (0.46–0.87) and TVS 0.68 (0.44–0.86) (*P* = .66); specificity: MR 0.86 (0.76–0.93) and TVS 0.65 (0.50–0.77) (*P* = .03)). They point out that MR diagnostic accuracy improves when excluding uteri >400 mL and conclude that the combination of MRI and TVS produces the highest level of accuracy for exclusion of adenomyosis. In addition, measurement of the difference in junctional zone thickness may optimize the MR diagnosis. In the study by Dueholm et al., the combination of MRI and TVS was most sensitive (0.89 (0.64–0.98)), but produced the lowest specificity (0.60 (0.44–0.73)). Exclusion of uteri >400 mL from the analysis improved the diagnostic precision of MRI, but not that of TVS. The diagnostic accuracy at MRI was also improved by calculating the maximum difference between the thinnest and thickest junctional zone (JZdif) (i.e., > or = 5–7 mm).

The availability of noninvasive, imaging techniques enabling a preoperatory diagnosis of adenomyosis [12–15], have not only revolutionised treatment [16], they have renewed interest by the scientific community on an otherwise neglected condition, creating a flurry of research activities leading also to improved knowledge of the relationship between adenomyosis and endometriosis [17].

## 2. The Uterine Junctional Zone

The inner myometrium, or junctional zone myometrium, also called “archimetra” [18] possesses a specific characteristic that distinguishes it from other similar junctions in the human body: it lacks a recognisable protective layer or membrane, forcing endometrial glands into direct contact with the myometrium. MR, T2-weighted images of the uterus, display in healthy women of reproductive age three distinct layers [14]: (1) the endometrial mucosa or innermost stratum, providing a signal of high intensity; (2) the already mentioned, intermediate area immediately subendometrial, giving a signal of low intensity and named junctional zone myometrium; (3) an outer zone extending all the way to the serosal layer, or outer myometrium, with a medium-signal intensity.

Recently, a classification for adenomyosis has been proposed by Gordts et al. [6]: simple JZ hyperplasia (zone thickness ≥8 mm but <12 mm on T2-weighted images, in women aged 35 years or less); partial or diffuse adenomyosis (thickness ≥12 mm; high-signal intensity myometrial foci; involvement of the outer myometrium: <1/3, <2/3, > 2/3), adenomyoma (myometrial mass with indistinct margins of primarily low-signal intensity on all MR sequences). Unfortunately, this classification has never been debated or submitted to a consensus meeting and, therefore, remains to be validated.

Research carried out over the last two decades has now provided proper information on the nature and functions of the JZ. It has been shown that the zone undergoes cyclical changes in its thickness that mimic that of the endometrium and are characterised by maximum growth between days 8 and 16 [12], making it a hormone-dependent structure that governs uterine peristalsis outside pregnancy. During postmenopause, under suppression of ovarian activity with hormonal contraception, or following administration of gonadotropin releasing-hormone analogues (GnRH-A), the myometrial layers become indistinct on MR imaging, although use of hormone replacement therapy results in the reappearance of the typical zonal anatomy [19].

Transabdominal ultrasound imaging has now shown the presence in the myometrium of distinct contraction waves; this peristaltic activity originates exclusively from the junctional zone, while the outer myometrium remains quiescent. During the follicular and periovulatory phases, contraction waves have a cervicofundal orientation and their amplitude and frequency increase significantly towards the time of ovulation [20]. These waves are probably implicated in many aspects of the physiological reproductive process: endometrial differentiation [21], menstruation [22], sperm transport [23], and implantation [24]. Myometrial contractions have the ability to transport and preferentially direct microspheres placed in the vagina to mimic spermatozoa, towards the peritoneal opening of the tubes on the side of the dominant follicle [25]. During the luteal phase, uterine contractility decreases and myometrial contraction waves become short and asymmetrical, often running in opposing directions. This reduced activity may help the implantation process that, classically, takes place near the fundus and possibly facilitates local supply of nutrients and oxygen. In addition, in humans, interstitial and intravascular trophoblast invasion goes beyond the endometrium and involves the junctional zone, but not the outer myometrium [26]. Finally, 7 days after ovulation, at a time coinciding with embryo implantation there is a focal disruption of the junctional zone signal intensity [24].

Given the fact that the presence of adenomyosis involves alterations of the myometrium, as well as of the ZJ, a critical area for successful reproduction, it seems reasonable to hypothesise the existence of a relationship with subfertility [27]. Evidence is also available of a close relationship between the occurrence of adenomyosis and the structural and functional defects in the eutopic endometrium and the myometrial uterine JZ. These abnormalities in turn may cause implantation failure and infertility [28].

## 3. Evidence Linking Adenomyosis to Infertility

As already stressed, the advent of high resolution imaging techniques has completely revolutionised our ability to identify the presence of milder forms of adenomyosis and, therefore, to explore a possible link with infertility.

Although no epidemiologic evidence exists, indirect data are available and provide a good case for an association between adenomyosis and infertility. Already fifteen years ago, de Souza et al. [27] reported an incidence of 54% myometrial JZ hyperplasia (a clear sign of adenomyosis) in subfertile patients complaining of menorrhagia or dysmenorrhoea. The mean age of these women was 34 years and some 70% of them were nulliparae. Several studies have confirmed the early work of de Souza: the disease can be present even in young women and be associated with both pelvic endometriosis and infertility and therefore may well represent a contributing factor [29–32]. This is more so since today, in western countries, an increasing number of women delay their first pregnancy until their late 30 s or early 40 s and, as a consequence, more women are found to have adenomyosis in fertility clinics during their diagnostic work-up [33].

Some evidence of an association can also be derived from reports of infertile women achieving pregnancy after being treated for adenomyosis. The first agents utilised for this purpose were GnRH-A [34] and several case reports or small series have been published with the analogue given alone, or in combination with surgery. In this connection, it has been found that, in IVF cycles, MR evaluation of junctional zone thickness is the best predictive factor of implantation failure [35], in the sense that an increase in JZ diameter is inversely correlated to the implantation rate. In fact, a thickened JZ is an independent factor for embryo implantation failure, and it is especially independent from embryo quality, infertility subtype, or patients age [36]. This observation has important clinical implications: in the presence of JZ thicker than 10 mm it becomes necessary to discuss with the patient whether to proceed immediately with IVF, or to postpone the procedure and carry out treatment with a GnRH analogue, a procedure that has the potential to reduce JZ thickness as assessed by successive MR [37]. Early results [38] seem to confirm an improvement of IVF results after this kind of therapy. In addition, prolonged pretreatment with GnRH-A before IVF has been reported to improve clinical pregnancy rates in infertile women with endometriosis [39]. Although, no data are available on women with adenomyosis, it seems reasonable to infer that also in this case pre-treatment may be beneficial.

Analogues can offer many advantages as a treatment for adenomyosis-associated infertility, over and above the hypooestrogenic state they produce: therapy with GnRH-A decreases expression of aromatase cytochrome P450 in the eutopic endometrium of women with adenomyosis and endometriosis [40] and it is well known that this enzyme is overexpressed in patients with these conditions. In women with adenomyosis, GnRH-A can suppress the generation of peroxynitrite, a compound known to cause tissue injury [41].

Several reports exist on the use of GnRH-A in the treatment of adenomyosis-associated infertility; the first case, ending in miscarriage, dates back to 1993 [42]; this was followed in 1994 by the first report of a successful term pregnancy [43]. Reports of small series of successful combined (GnRH-A plus surgery) treatment in women with adenomyosis seeking pregnancy have also appeared [44–47]. Additional evidence of a linkage between adenomyosis and infertility comes from a small Japanese study using an intrauterine system releasing danazol, in which three out of four infertile women conceived after removal [48]. A second, more recent option is offered by the levonorgestrel-releasing IUS, known as Mirena, although—so far—it has been only utilised for the relief of symptoms associated with adenomyosis [49, 50]. Finally, surgery has also been utilised to restore fertility in women with adenomyosis; a new conservative surgical technique called “adenomyomectomy” seems to offer good results (a pregnancy rate of around 50%) [51].

On a different front, there is good experimental evidence linking adenomyosis to infertility. Indeed, in baboons adenomyosis is not only strongly associated with lifelong primary infertility, but also statistically significantly associated to endometriosis [52]. Finally, it has been known for some times that a subfamily of homeobox genes named Abdominal B (*AbdB*), are involved in the developing urogenital system in vertebrates. Satokata et al. [53] have mutated one of the *AbdB* genes named *Hoxa10* in mice and observed that female homozygotes ovulate normally, but—if pregnancy occurs—in the great majority of animals it ends with the death of all embryos, and abortion occurs at the time when the *Hoxa10* gene should be expressed (2.5 to 3.5 days after coitus). This means that proper expression of the maternal *Hoxa10* gene is necessary to maintain viability of the preimplantation embryo, and, recently, it has been proven that in women with adenomyosis the expression of *Hoxa10* gene is decreased during the secretory phase of the cycle, a possible explanation for the observed lower implantation rate in women with adenomyosis [54].

## 4. Possible Mechanisms Involved in Adenomyosis-Associated Infertility

The above-mentioned data not only support the hypothesis that adenomyosis may be associated with infertility; they also provide a number of clues as to which mechanisms may be involved. Indeed, structural and functional defects of the uterine JZ, as well as the existence of several dysregulated proteins can cause implantation failure. In addition, a number of other conditions can, in theory at least, impair fertility: the presence of abnormal levels of intrauterine free radicals; an aberrant endometrial development throughout the menstrual cycle, possibly as a consequence of an abnormal local steroid metabolism; a lack of expression of some of the “implantation markers”; an altered function of genes essential for embryonic development.

### 4.1. Dysregulation of Myometrial Architecture and Function

An interesting comparative analysis of protein expression in adenomyotic tissue and in normal myometrium has been conducted by Liu et al. [55], who found that in women with adenomyosis there are 12 dysregulated protein spots and were able to identify 10 of them by mass spectrometry. In subjects with adenomyosis myocytes exhibit cellular hypertrophy, to the point that smooth muscle cells become ultrastructurally different from smooth muscle cells of normal uteri. The JZ shows cellular and nuclear hypertrophy, abnormal nuclear and mitochondrial shape, and a number of other abnormalities that may cause a disturbance in the normal calcium cycling in the affected myocytes, with a subsequent loss of normal rhythmic contractions [56]. Although it is too early to conclude that these phenomena may be implicated in creating a subfertility condition, it has been shown that adenomyosis causes an impairment of the rapid, sustained, and accurately directed sperm transport in the uterus consequent to the destruction of the normal architecture of the JZ myometrium [18]. These patients also show a reduced uterotubal transport capacity that progressively decreases with increasing severity of the disease; also, a major disruption of uterotubal transport has been detected using radionuclides in women with diffuse adenomyosis and primary infertility [57, 58]. Finally, adenomyosis is associated to a loss of nerve fibres at the endometrial-myometrial interface [59].

Although no definite explanation exists for the role of a thickened JZ in reducing implantation rates, the hypothesis has been brought forward that, under abnormal hormonal influence, ectopic endometrial glands can trigger an “inflammatory” reaction. This would be mediated by cytokines, prostaglandins, or other still unspecified factors and would determine smooth muscle proliferation that, in turn, would alter uterine contractions [60].

### 4.2. Altered Endometrial Function and Receptivity

Within the endometrium itself, the presence of abnormal levels of free radical concentration represents a possible cause for infertility in adenomyosis patients. This is because a disruption of the balance between reactive oxygen species and antioxidants produces oxidative stress and an excessive free radical environment. In turn, this can damage fertilized eggs and inhibit embryo development and pregnancy, and Noda et al. [61] have shown that low concentrations of free radicals are necessary to create an appropriate environment for early embryonic development. In the presence of abnormal levels of free radicals the embryo may be attacked by activated macrophages or T cells, or be exposed to an excess of nitric oxide, which may result in early miscarriage [62]. A number of investigations have focused on enzymes producing or eliminating free radicals: two of them are particularly interesting in this context: xanthine oxidase (XO) that produces superoxide and superoxide dismutase (SOD) that eliminates it, while simultaneously producing hydroxyl radicals, that, in turn, can be eliminated by glutathione peroxidase (GPx). It has been shown that in women with adenomyosis, nitric oxide synthase (NOS), XO, SOD, and catalase levels do not fluctuate and are over expressed [63, 64]; interestingly, as already mentioned, administration of GnRH-A suppresses the expression of both eNOS and iNOS and the formation of peroxynitrite in adenomyosis [40].

Altered oxidative stress equilibrium is not the only mechanism through which a uterine environment hostile to the developing embryo can be produced in women with adenomyosis. Another important abnormality that may lead to an impairment of implantation has now been identified: in women with adenomyosis there is an aberrant endometrial development throughout the proliferative phase, and this may lead to abnormalities of the secretory phase. This seems due to altered endometrial vascularisation, an increase in regulatory factors involved in the endometrial vascular proliferation and changes in endometrial molecular markers of inflammation [65, 66]. Indeed, in subjects with adenomyosis, in both eutopic and ectopic endometria there is a significantly greater activity of the vascular endothelial growth factor (VEGF) of microvessel density [67] and of the hypoxia-inducible factor-1alpha [68]. Furthermore, a series of anomalies have been found in the secretion of interleukins in both eutopic and ectopic endometria of subjects with adenomyosis, again leading to a disruption of early events related to implantation. These anomalies involve an improper secretion of interleukins-6 [69], -8 [65], and -10 [70]. In conclusion, in women with adenomyosis an abnormal inflammatory response seems to exist and impair nidation.

There is a third mechanism through which an altered endometrium can lead to implantation failure: an abnormal intraendometrium metabolism. Since in adenomyosis IL-6 is over expressed [69], this could lead to increased oestrogen receptor expression and, indeed, the expression of the different isoforms of oestrogen receptor alpha (ER-*α*) and beta (ER-*β*) and progesterone receptor A (PR-A) and B (PR-B) are differentially modulated in uteri with adenomyosis compared with controls [71]. In addition, in the endometrium of subjects with adenomyosis there is over expression of cytochrome P450 [72]; this phenomenon increases local oestrogen production [73], and it has been shown that an over expression of endometrial aromatase significantly lowers clinical pregnancy rates (with similar numbers of retrieved oocytes and replaced embryos with respect to controls) [74]. In these women there is also a defect in progesterone receptors and loss of their action [75]; this altered balance between oestrogen and progesterone results in the persistence of ER-*α*, given that downregulation of this receptor is one of the primary functions of progesterone. The overexpression of ER-*α* in midsecretory phase reduces the secretion of beta 3 integrins, negatively regulated by oestrogens, thereby altering uterine receptivity [76]. The observed reduction in PR expression may even explain the poor response to progestational agents in women with adenomyosis [77].

In adenomyotic foci, ER-*α* staining does not vary during the menstrual cycle in either glands or stroma; conversely, there are no cyclical changes in its expression in the innermost or outer myometrium. Furthermore, ER-*β* expression in the proliferative phase is statistically significantly higher in the *functionalis* portion of the glands compared with controls. Expression is similarly higher in the *basalis*, the stroma, the JZ, and outer myometrium, compared with control tissue where expression is weak and shows no statistically significant variation with the phase of the cycle [71]. The higher ER-*β* expression in the myometrium of adenomyotic uteri might thus contribute to the presence of the classically described myometrial hyperplasia [55].

A fourth mechanism that can lead to implantation failure is a lack of expression of some of the molecules, labelled “implantation markers,” that are expressed by the endometrium and are required for the successful interaction between embryo and endometrium. In 2006, Yen et al. [78] have reported that during the implantation window, some of these markers are decreased in the endometrium of women with adenomyosis, suggesting that this may be one of the molecular mechanisms associated with a decreased implantation rate.

In particular, it has been demonstrated that the so-called Leukemia Inhibitory Factor (LIF) is associated with endometrial receptivity and is lower in women with infertility compared with healthy controls [79]. It has also been shown that LIF expression is decreased in the endometrium in women with adenomyosis during midsecretory phase and, when these women have a history of infertility, they show significantly lower LIF levels in uterine flushing fluid, compared with fertile controls [80].

One of them, the *α*-4, *β*-3 integrin appears on the surface of epithelial cells of both embryo and endometrium and on maternal surfaces around cycle day 19 to 20 and continues to be expressed during pregnancy [81]. Although it is not known whether its expression is modified in women with adenomyosis, it has been shown that integrin is missing in a subset of women with unexplained infertility and endometriosis [82]. Information on this and several other proteins such as glycodelin, osteopontin, and vitronectin that are believed to mediate trophoblast-endometrial interactions during implantation and are downregulated in women with endometriosis [83, 84], is still lacking in the case of adenomyosis, but it can at least be speculated that a mechanism of this kind may also be involved.

A fifth important factor that may be involved in creating an impairment of implantation in women with adenomyosis is the already mentioned altered function of the HoxaA10 gene. As stated above, this gene is part of a homeobox-containing transcription factors essential for embryonic development and proper adult endometrial growth during the menstrual cycle [85] and in women with adenomyosis expression its is significantly lower during the midsecretory phase compared with fertile controls [54].

## 5. Conclusions

At present it is impossible to show conclusively that adenomyosis can lead to subfertility or infertility because no epidemiologic studies have ever been carried out. At the same time, it is hoped that the introduction of MR and, even more, that of the more readily available 3D-TVS will facilitate early diagnosis and help collecting missing data. Notwithstanding this unsatisfactory situation, a careful look at molecular pathophysiology of the disease and at preliminary clinical results with a number of new techniques [28] can already help clarifying the situation although, the final answers lie in the execution of controlled clinical investigations.
